# Filamentous actin accumulates during plant cell penetration and cell wall plug formation in *Phytophthora infestans*

**DOI:** 10.1007/s00018-016-2383-y

**Published:** 2016-10-06

**Authors:** Kiki Kots, Harold J. G. Meijer, Klaas Bouwmeester, Francine Govers, Tijs Ketelaar

**Affiliations:** 1 Laboratory of Phytopathology, Wageningen University, Droevendaalsesteeg 1, 6708 PB Wageningen, The Netherlands; 2 Laboratory of Cell Biology, Wageningen University, Droevendaalsesteeg 1, 6708 PB Wageningen, The Netherlands; 3 Plant-Microbe Interactions, Department of Biology, Utrecht University, Padualaan 8, 3584 CH Utrecht, The Netherlands; 4 Business Unit Biointeractions and Plant Health, Wageningen University and Research, Wageningen Plant Research, Droevendaalsesteeg 1, 6708 PB Wageningen, The Netherlands

**Keywords:** Actin cytoskeleton, Appressorium, Oomycete, Late blight, Plant pathogen, Lifeact

## Abstract

The oomycete *Phytophthora infestans* is the cause of late blight in potato and tomato. It is a devastating pathogen and there is an urgent need to design alternative strategies to control the disease. To find novel potential drug targets, we used Lifeact-eGFP expressing *P. infestans *for high resolution live cell imaging of the actin cytoskeleton in various developmental stages. Previously, we identified actin plaques as structures that are unique for oomycetes. Here we describe two additional novel actin configurations; one associated with plug deposition in germ tubes and the other with appressoria, infection structures formed prior to host cell penetration. Plugs are composed of cell wall material that is deposited in hyphae emerging from cysts to seal off the cytoplasm-depleted base after cytoplasm retraction towards the growing tip. Preceding plug formation there was a typical local actin accumulation and during plug deposition actin remained associated with the leading edge. In appressoria, formed either on an artificial surface or upon contact with plant cells, we observed a novel aster-like actin configuration that was localized at the contact point with the surface. Our findings strongly suggest a role for the actin cytoskeleton in plug formation and plant cell penetration.

**Electronic supplementary material:**

The online version of this article (doi:10.1007/s00018-016-2383-y) contains supplementary material, which is available to authorized users.

## Introduction

*Phytophthora infestans* is a plant pathogen in the class oomycetes, filamentous organisms that resemble fungi in lifestyle and morphology but without evolutionary relationship with fungi. Oomycetes belong to the Stramenopile lineage together with the brown algae and diatoms [1] and are well-known as pathogens mainly of plants but also of animals and other organisms. The genus *Phytophthora* comprises over 120 species, many of which are devastating plant pathogens [2]. *Phytophthora infestans,* the causal agent of potato late blight, is the most notorious one and famous since the Great Irish Famine in the mid-nineteenth century. Today, *P. infestans* is still a major problem for potato production worldwide. For controlling late blight farmers spray crop protection agents every 5–7 days and up to 17 times per growing season. Similar intensive chemical treatments are needed to control other oomycete pathogens, not only in crops but also in aquaculture where saprolegniasis, a disease caused by *Saprolegnia parasitica,* is a major problem in salmon farming [3].

Oomycetes grow as mycelium and reproduce and disperse by means of spores. The vegetative propagules of *P. infestans* are sporangia that germinate directly or indirectly, depending on the ambient temperature. At temperatures lower than 15 °C the sporangia cleave and release motile zoospores, while at higher temperatures the sporangia can germinate directly [4, 5]. When encountering a suitable environment, like a leaf surface, the hyphal germlings emerging from sporangia or from encysted zoospores develop an appressorium at the tip, and subsequently a penetration peg is formed that pierces the plant epidermis. After the pathogen has gained access to the plant, the hyphae grow intercellular in the mesophyll occasionally forming digit-like structures called haustoria that penetrate plant cells [4, 5]. Contrary to fungal hyphae, the hyphae of oomycetes lack septa or cross walls and are therefore referred to as aseptate or coenocytic. However, under certain circumstances septa, in some cases referred to as cross walls, have been observed in oomycetes, for example at the basis of the sporangium, at the hyphal tip, in old mycelium or in response to wounding [6–8]. Interestingly, in *P. infestans* septa-like structures have also been described to form in the germ tube, separating the cyst from the appressorium [9].

Actin is an essential structural component in eukaryotic cells [10]. The actin cytoskeleton that consists of a highly dynamic network of filamentous actin polymers (F-actin) is involved in many cellular processes, including muscle contraction, cell motility, cytokinesis, and vesicle and organelle transport [11–13]. The precise function of the actin cytoskeleton differs among organisms and between tissues. For example, in tip-growing organisms such as fungi and oomycetes, and also in pollen tubes and root hairs, the actin cytoskeleton is indispensable for establishing and maintaining tip growth [14–16]. In oomycetes, F-actin is organized in two prominent higher order structures, namely actin cables and dot-like actin structures, called actin plaques. Additionally, a few oomycete species, i.e., *Saprolegnia ferax*, *Achlya bisexualis,* and *Phytophthora cinnamomi*, were shown to have hyphae with an apical cap of F-actin [14, 17, 18]. Actin plaques seem to occur at higher frequencies in non-growing parts of the hyphae and in resting structures, while actin cables are more abundant during germination and in growing hyphae [19–21]. Moreover, as was found in *P. infestans,* plaques are more resilient to the actin depolymerizing drug latrunculin B than cables [20, 21]. The function of the different actin structures in oomycetes remains elusive. Previously it was hypothesized that actin plaques in oomycetes are similar to actin patches in fungi, with the latter functioning as force generators for vesicle internalization during endocytosis [11, 22–25]. However, our recent study in which we used fluorescently tagged Lifeact for live cell imaging of the actin cytoskeleton in *P. infestans* showed that actin plaques in *P. infestans* have a far longer lifetime and are much less mobile than actin patches in fungi [21]. We also showed that, in contrast to patches, plaques are not internalized and therefore it is unlikely that plaques have a function in endocytosis.

Prior to host cell invasion many (hemi-)biotrophic filamentous plant pathogens, including *P. infestans,* form a specialized rigid infection structure known as appressorium that facilitates penetration of the host. Microscopic imaging of the actin cytoskeleton in a Lifeact-RFP expressing line of the rice blast fungus *Magnaporthe oryzae* revealed that during plant cell invasion a toroidal F-actin network, scaffolded by septins, is assembled in appressoria [26]. Septins are small guanosine triphosphatases (GTPases) that are involved in reorientation and reorganization of the cytoskeleton. In this study, we exploited the previously described *P. infestans* Lifeact-eGFP strains [21] to investigate the organization and dynamics of the actin cytoskeleton in *P. infestans* in germ tubes emerging from sporangia or cysts, and during appressorium formation and plant cell infection. For this purpose we used two culture conditions. On the one hand, we allowed sporangia or cysts to germinate on a hydrophobic surface that triggers the formation of appressoria in the absence of the host plant and; on the other hand, we used a so-called in vitro infection system that makes use of tomato MsK8 cells grown in suspension. In this system, we can mimic leaf infection and take advantage of the fact that the infection process is more synchronized and more suitable for microscopic imaging. In addition to the cortically localized actin cables and actin plaques that we described previously [21, 27], we identified two novel actin configurations. The first one is an actin accumulation in appressoria, at the site of contact with the hydrophobic surface or, in the case of the in vitro infection system, at the site where the penetration peg emerges from the appressorium to enter the host cell. The second one is associated with a structure that divides the germ tube into two compartments, one toward the tip that is full of cytoplasm and a basal part that seemingly lacks cytoplasm. We show here that this basal part is sealed off by cell wall plugs that are deposited centripetally and that this plug formation is preceded by a localized accumulation of actin filaments that remain present until a plug has been deposited. These observations provide further insights into the organization of the actin cytoskeleton in *P. infestans*, in particular during cyst germination and in appressoria, the structures that play an important role in pathogenesis.

## Materials and methods

### Strains and cultures

*Phytophthora infestans* strain 88069 and a Lifeact-eGFP (previously described in [21]) expressing strain were maintained on Rye Sucrose Agar plates (RSA [28]), with appropriate selective antibiotics as described in Meijer et al. [21]. Cultures were maintained by transferring mycelial plugs from fully grown plates to fresh plates every 2–3 weeks. The tomato MsK8 cell suspension culture was grown as described by Koornneef et al. [29].

### Sample preparation

Zoospores were released by flooding sporulating cultures with Milli-Q water (4 °C) and subsequent incubation at 4 °C for 3 h. The zoospore suspension was filtered through Miracloth (Calbiochem, Germany) and the zoospores were encysted by vortexing the filtrate for 5 min. Sporangia were collected as described previously [21]. The cysts or sporangia were then transferred to bio-foil slides [21] containing water or liquid RS medium and incubated for 5 h at 18 °C to allow cyst germination [21]. For plant cell infection assays, 2 ml of zoospore suspension (4 × 10^6^ zoospores per ml) was added to 2 ml of a 5 days old tomato MsK8 cell suspension culture (C. Schoina, personal communication). This mixture was incubated overnight at room temperature on a rotary shaker (80 rpm) in the dark. Alternatively, a small fraction of the plant cell and zoospore mixture was enclosed in a bio-foil slide and incubated stationary overnight at room temperature in the dark.

### Microscopy

To visualize F-actin dynamics a Roper Spinning Disk Confocal System (Evry, France) consisting of a CSU-X1 spinning disk head (Yokogawa, Japan) mounted on a Nikon Eclipse Ti microscope (Tokyo, Japan) with Perfect Focus system was used. For imaging we used a 100× Plan-Apochromat 1.4 N.A. oil immersion objective. Fluorescence imaging was performed using a 491 nm laser line combined with band pass emission filtering (530/50 nm; Chroma Technology). Images were acquired with an Evolve electron-multiplying (EM) charge-coupled device camera (Photometrics) at an EM gain of 200, controlled by Metamorph software (Molecular Devices, California). *Z*-stacks were collected using a 0.5 μm step size.

For cell wall staining, solutions with calcofluor white (Sigma-Aldrich, Missouri) or aniline blue (Sigma-Aldrich, Missouri) were added to germinated cysts shortly before imaging to the final concentrations of 0.0017 and 0.2 % w/v, respectively. Fluorescence imaging was performed using a Zeiss LSM 510 Meta confocal microscope equipped with a 63× Plan-Apochromat 1.4 N.A. oil immersion objective. Calcofluor white and aniline blue were imaged using the 405 nm laser line combined with a 420–480 nm band pass emission filter and a 420 nm long pass filter, respectively.

### Image processing

All image processing was carried out using the software package ImageJ (rsbweb.nih.gov/ij/). Collected images were enhanced by contrast stretching and maximal intensity *Z*-projections were made. Orthogonal views were constructed by scaling the *Z*-axis to the size of *x, y* pixel dimensions (Scale plugin) and making kymographs (Multiple kymograph plugin) of scaled *Z*-stacks.

## Results

### Germ tubes emerging from cysts have plugs containing cellulose and callose

**Fig. 1:**
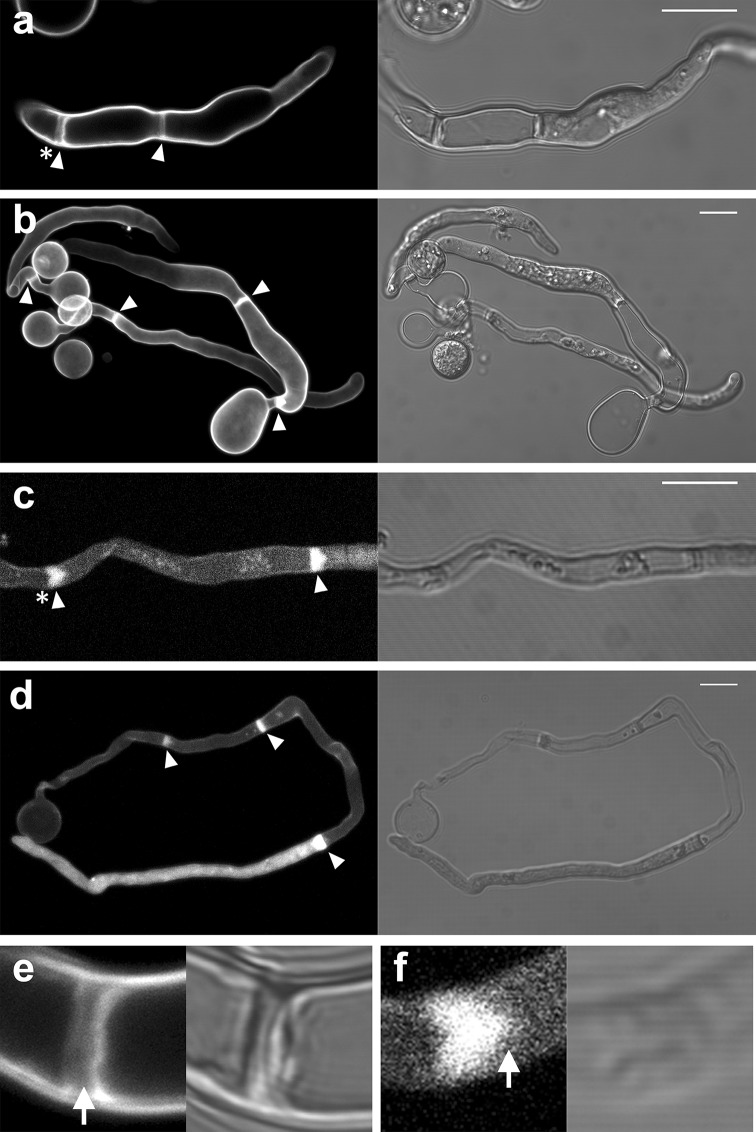
Plugs in germ tubes emerging from *P. infestans* cysts contain cellulose and callose. Plugs (*arrowheads*) are visualized by staining with calcofluor white (**a**, **b**, **e**) and aniline blue (**c**, **d**, **f**) that reveal cellulose and callose, respectively. The plugs indicated with the *asterisk* in **a** and **c** are shown in more detail in **e** and **f**, respectively. In **e** the *arrow* indicates the middle lamellae of the plug, which is not stained by calcofluor white. The *arrow* in **f** indicates the protruding side of the plug pointing toward the tip. The images represent single confocal planes (**a**, **c**, **e**, **f**) or *Z*-projections (**b**, **d**). *On the right* are the bright field images and *on the left* the fluorescent channel images. *Bars* 10 μm

When a zoospore touches a solid surface it immediately encysts and starts to germinate. In this early stage the cyst and germ tube are filled with cytoplasm. However, we noticed that upon germ tube elongation, the majority of the cytoplasm relocates to the tip of the germ tube, a phenomenon that has been described before [9]. After this retraction of cytoplasm from the cyst and basal part of the germ tube, we observed the formation of a wall-like structure that separated the basal part depleted of cytoplasm from the cytoplasmic rich apical part of the hypha (Fig. 1). This wall-like structure is reminiscent of structures observed in pollen tubes that are referred to as callose plugs. Callose plugs in pollen tubes, like the wall-like structure in *P. infestans,* separate the cytoplasm-filled tip from the empty basal part [30]. In oomycete literature the term plug has been used to describe the cell wall material that is deposited in response to impalement with a glass electrode [6], the term cross wall has been used to describe the wall-like structure that separates a sporangium from a hypha [6] and the term septum has been used to describe cross walls in hyphae [7]. Since the structures that we observe in the germ tubes are not associated with cytokinesis but appear to seal off a compartment that is depleted of cytoplasm, we hereafter use the term cell wall plug or simply plug.

**Fig. 2:**
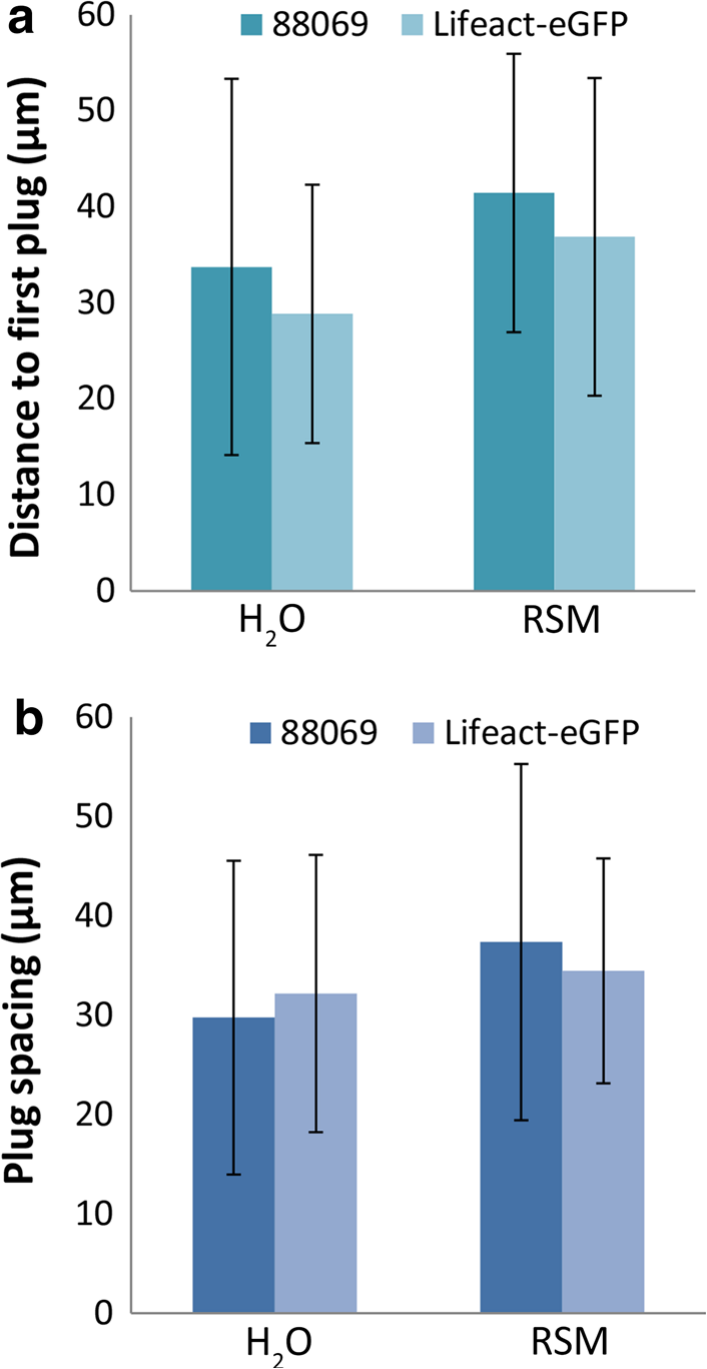
The distance of the hyphal tip to the first deposited plug (**a**) and the spacing between plugs (**b**) in hyphae emerging from cysts from *P. infestans* strain 88069 and the Lifeact-eGFP expressing derivative. Per data point 9–26 hyphae were measured. *Error bars* indicate standard deviations

To determine the influence of nutrients on cyst germination rates and plug formation we compared nutrient poor (water) with nutrient rich (rye sucrose medium; RSM) growth conditions. During overnight incubation of cysts of strain 88069, the germination rate in water and RSM was comparable and in most hyphae multiple plugs were deposited (up to 6 per hyphae) with no significant difference between water and RSM [*p* = 0.44, unpaired *t* test (*n* = 46)]. The distance between the first deposited plug and the base of the cyst was on average 38.1 µm and the distance between plugs 33.7 µm with no significant differences between water and RSM [Fig. 2a, b; *p* = 0.12, unpaired *t* test (*n* = 46) and *p* = 0.21, unpaired *t* test (*n* = 32), respectively]. We also tested whether the expression of the Lifeact-eGFP caused differences in plug formation and positioning by comparing the previously described Lifeact-eGFP expressing line [21] with its recipient strain 88069. No significant differences were found between strains in the distance between plugs or the distance between the first plug and the cyst (Fig. 2; unpaired *t* tests, *n* > 20 and *p* > 0.11 for all tests).

To learn more about the composition of the plugs, we stained hyphae with calcofluor white, which emits fluorescence when bound to chitin and cellulose [31] and aniline blue, which has a high specificity for callose [32, 33]. Since oomycetes have hardly any chitin in their cell walls (<1 % of the cell wall carbohydrates [34–36]), the calcofluor white fluorescence most likely represents cellulose. Figure 1 shows that staining with either calcofluor white or aniline blue resulted in a strong fluorescent signal, but the localization of the fluorescence differed. In 50 % (*n* = 24) of the plugs that were studied by confocal microscopy, calcofluor white staining revealed two lamellae with a high fluorescence intensity that surrounded a lamella with a much lower fluorescence intensity (detail shown in Fig. 1e). No such layering was observed in plugs in the aniline blue-stained hyphae (detail shown in Fig. 1f; *n* = 24). Instead, the aniline blue-stained plugs were often (50 %) triangularly shaped, with the protruding side of the plug pointing towards the hyphal tip (Fig. 1f). Occasionally, in the calcofluor white-stained plugs a similar small protrusion pointing towards the hyphal tip could be observed. The layering that was often observed in the calcofluor white-stained plugs and never in the aniline blue-stained plugs, suggests that unlike callose, cellulose is not evenly distributed in plugs.

### A ring-shaped actin filament assembly correlates with plug formation

**Fig. 3:**
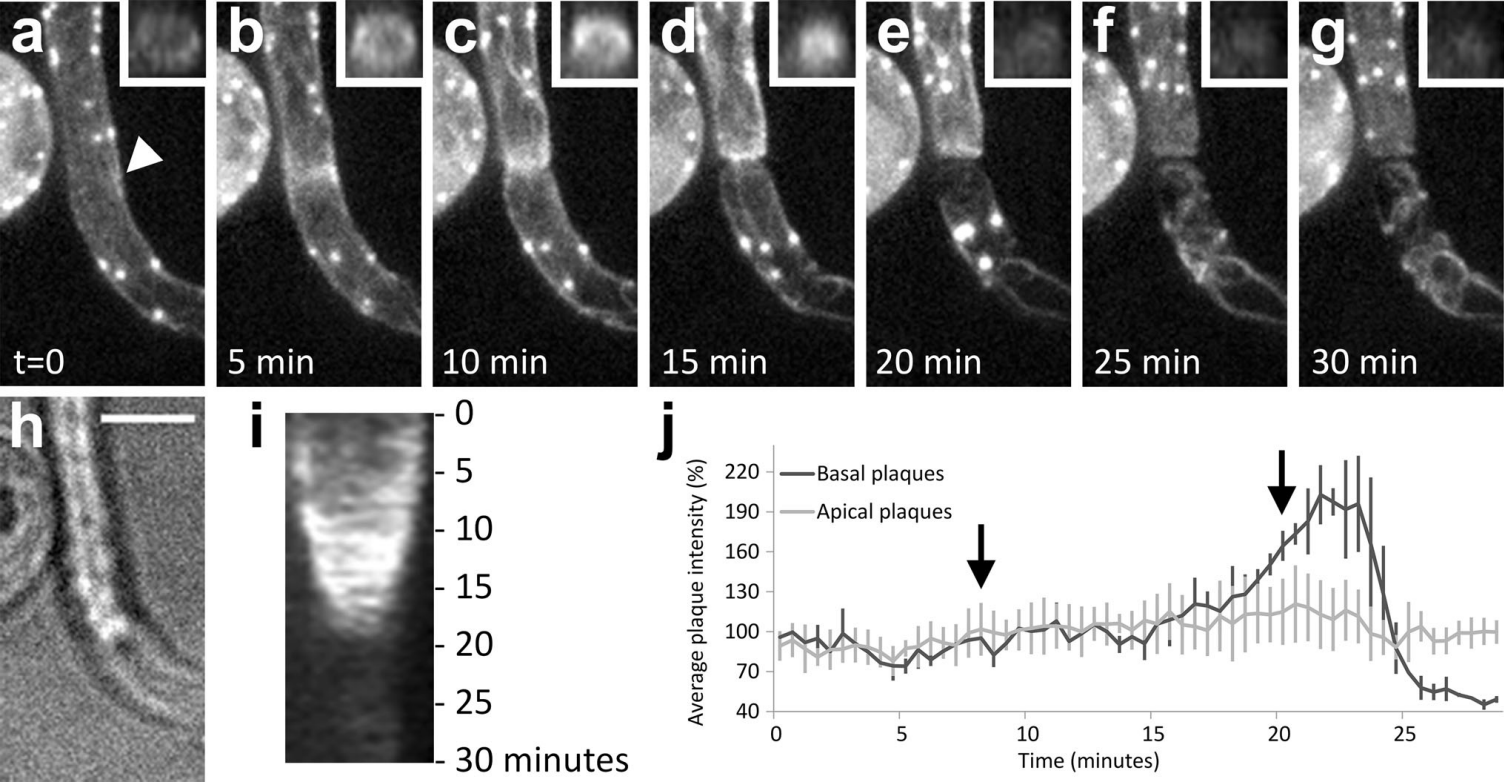
A local accumulation of actin filaments precedes plug formation and is associated with the expanding plug during plug deposition. Plug formation over time (**a–g**) visualized by maximum intensity projections of *Z*-stacks of a hypha of a Lifeact-eGFP expressing *P. infestans* strain with the hyphal tip (not visible) pointing upwards. The *arrowhead* in **a** indicates the plug deposition site. The *insets* in **a**–**g** are orthogonal views of the *Z*-stacks at the site where the plug is deposited. **h** is a bright field image of **d**. **i** Kymograph of the plug deposition. **j** Average fluorescence intensity in percentage (*Y*-axis) over time of the plaques that remain in the basal part of the hypha after plug formation (*n* = 3, *dark gray line*) with the average intensity over time of randomly picked plaques in the apical part of the hypha (*n* = 7, *light gray line*) set to 100 %. At *t* = 8 (*first arrow*) plug deposition is first visible and at *t* = 20 plug formation is completed (*second arrow*). *Error bars* indicate standard deviations. *Bar* 5 μm

**Fig. 4:**
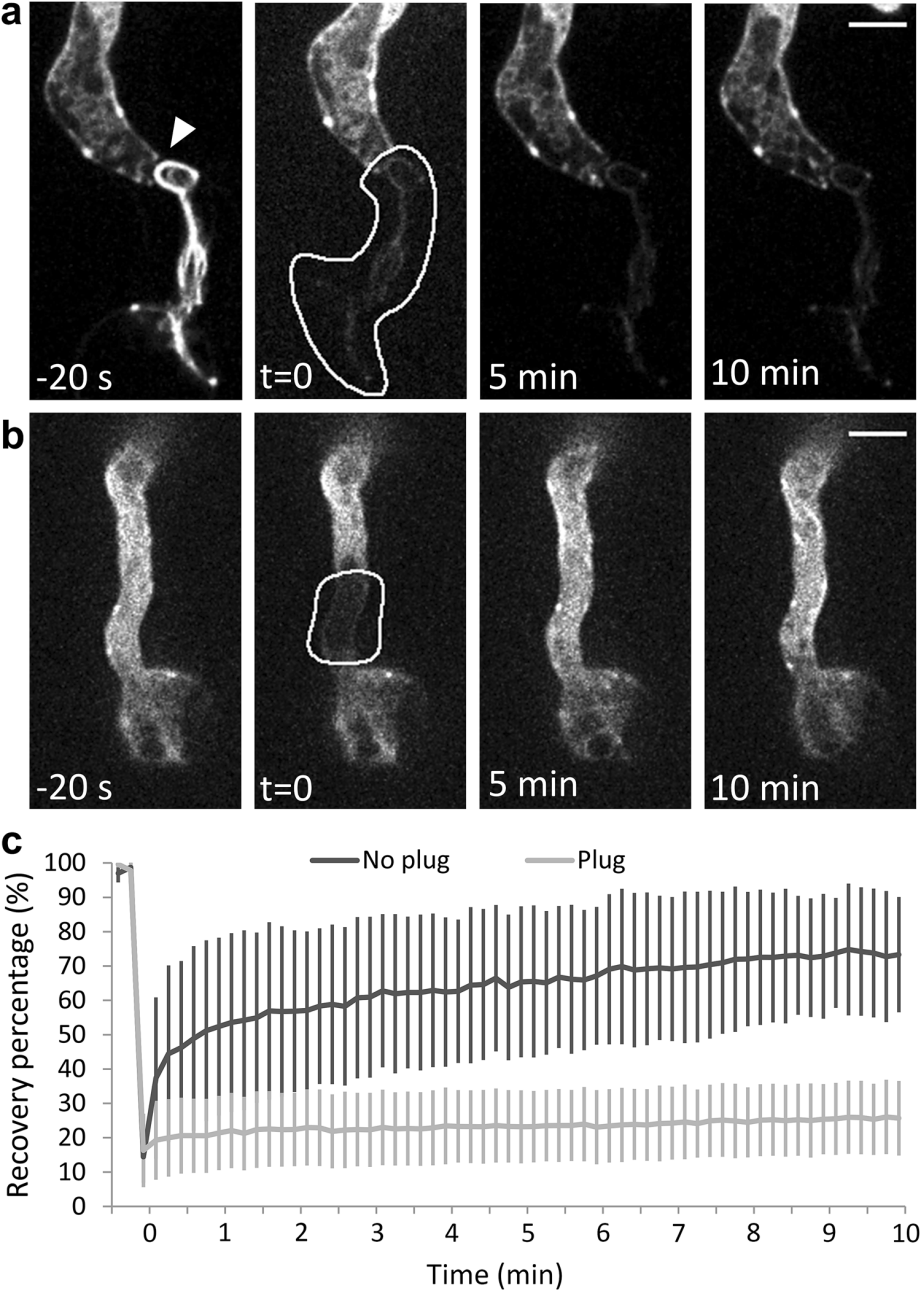
Plugs in a *P. infestans* hypha block the cytoplasmic streaming between the apical and basal part of the hypha. A compartment of the hypha that was sealed off by a plug (**a**; indicated by the *arrowhead*) and a part of the hypha that was in contact with the rest of the hypha (**b**) was photobleached and recovery of Lifeact-eGFP was recorded over 10 min. In **a** and **b** *panels* marked with −20 s show the fluorescence before photobleaching and *t* = 0 shows the area selected for photobleaching (*white line*). Recovery was shown immediately after photobleaching (*t* = 0) and after 5 and 10 min. *Bars* 5 µm. **c** The average fluorescence recovery after photobleaching (*Y*-axis) over time (*X*-axis) for hyphae with plug (*n* = 7) and hyphae without plug (*n* = 8). Recovery was monitored every 10 s

Next, we studied the actin configuration during plug formation. Although most Lifeact-eGFP is bound to filamentous actin, a limited amount of fluorescence does not appear to co-localize with actin filaments. This is recognizable as a faint staining of the cytoplasm and nucleus. We used this fluorescence to study the cytoplasmic organization during plug formation by confocal microscopy. This revealed that plug formation is preceded by a rapid depletion of the cytoplasm from the basal part of the hypha (Video S1). During cytoplasmic depletion, most of the Lifeact signal disappeared together with the retracting cytoplasm. Notably, the actin plaques frequently started to migrate into the apical part of the hypha and subsequently, F-actin started to accumulate at the ultimate site of septum formation, resulting in a ring-like assembly (Fig. 3a–g ). At the site of the actin accumulation, plug formation was initiated by centripetal ingression of the Lifeact fluorescence while cell wall material was deposited. During the centripetal expansion of the plug, the F-actin remained associated with its leading edge. Plug deposition took on average 11.5 min (±1.9; *n* = 10). A kymograph displaying the kinetics of plug formation revealed a uniform extension velocity during plug deposition (Fig. 3i). After completion of the plug, a small amount of cytoplasm was left basal of the plug in which a limited number of actin cables and plaques were observed. This remnant of cytoplasm basal of the plug lacked a nucleus, but a limited number of actin cables were maintained; this is in contrast to plaques that rapidly disappeared upon plug completion (Fig. 3a–g). In ~50 % of the hyphae, the plaques in the cytoplasm remnant displayed a specific, rapid increase in intensity before fluorescence diminished and the plaques disappeared (Fig. 3a–g, j) After disappearance of the plaques, hardly any or no cytoplasmic streaming was observed (Video S2). To investigate if there is a continuum between the cytoplasm apical and basal of the plug, the Lifeact-eGFP in the compartment basal of the plug was photobleached and the recovery was monitored. In contrast to the controls, i.e., in hyphae lacking plugs, where we could observe recovery, there was hardly any recovery visible in compartment basal of the plug and it is therefore unlikely that there is cytoplasm streaming from the apical to the basal part of the plug or vice versa (Fig. 4).

### A novel aster-like actin configuration in appressoria

**Fig. 5:**
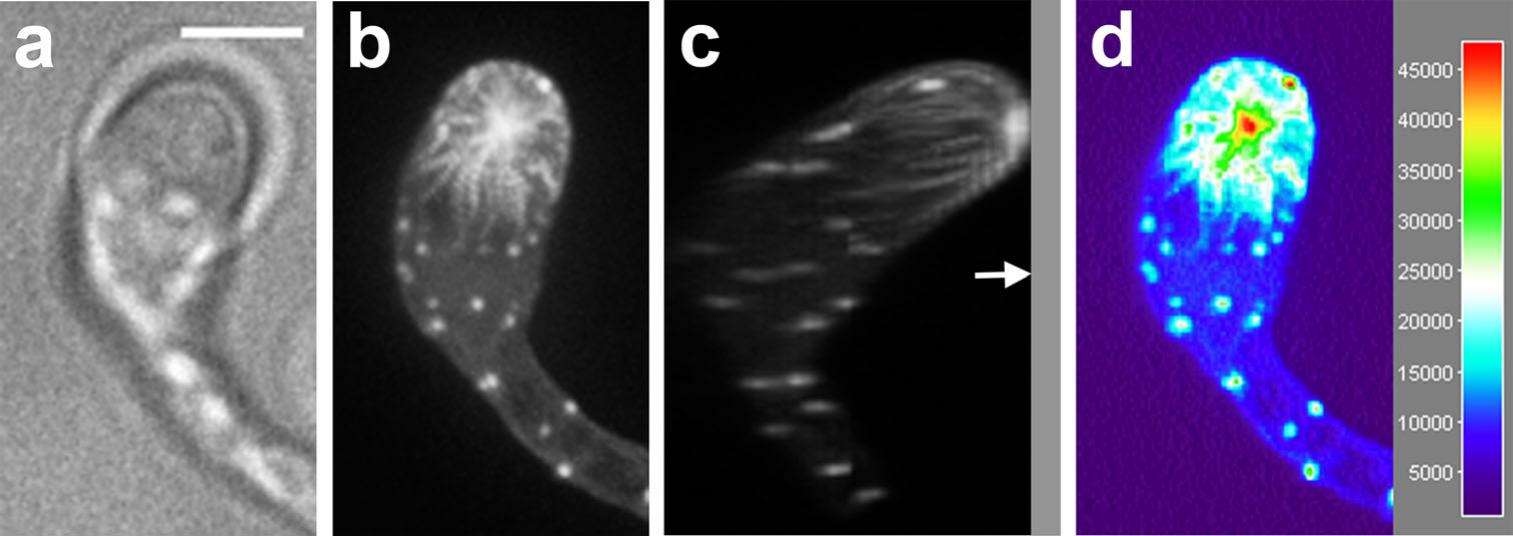
An appressorium of *P. infestans* with an aster-like actin structure at the contact point with the cover slip. Bright field image (**a**) and *Z*-projection showing the fluorescence of Lifeact-eGFP (**b**). **c** is a side view of **b**. The *arrow* points to a *gray-shaded bar* that represents the cover slip. **d** Surface plot showing the fluorescence intensity of **b**. *Bar* 5 μm

**Fig. 6:**
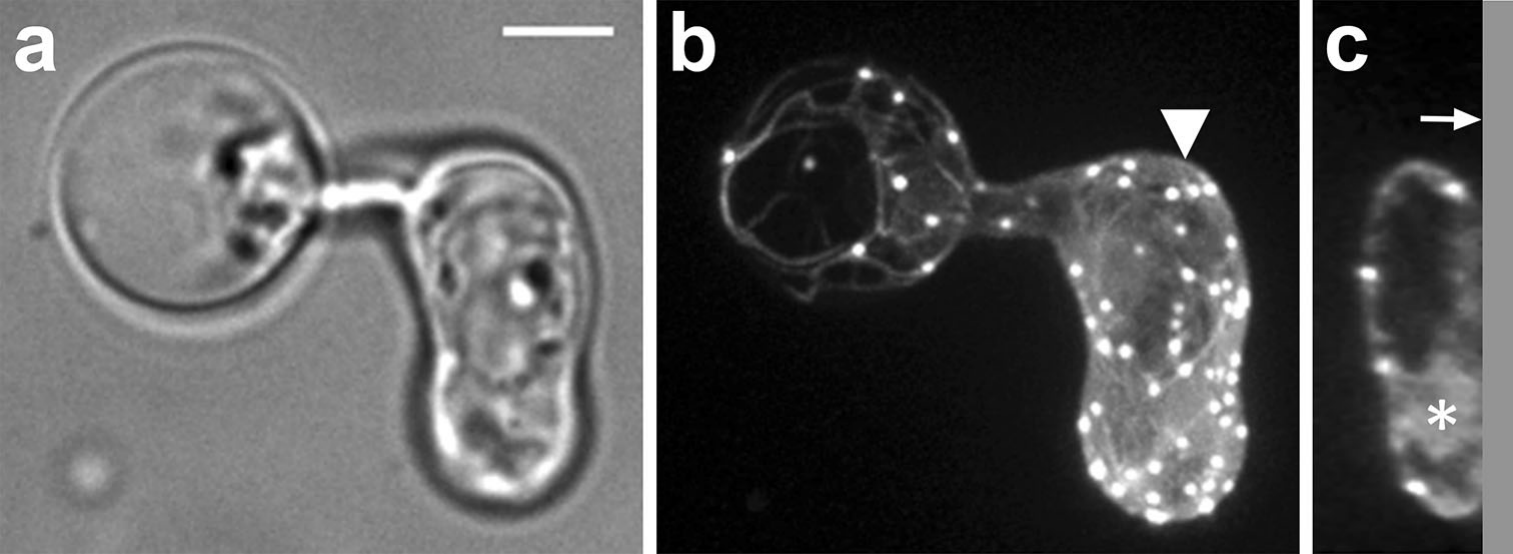
Localization of actin in an appressorium formed by swelling of the tip of a germ tube that is emerging from a *P. infestans* cyst and adheres to a solid surface. Bright field image (**a**) and maximum intensity *Z*-projection (**b**) showing the fluorescence of Lifeact-eGFP. The *arrowhead* in **b** indicates the position of the orthogonal projection shown in **c**. In **c** the *arrow* points to a *gray-shaded bar* that represents the cover slip and the *asterisk *marks the nucleus. *Bar* 5 μm

When a cyst germinates and encounters a plant surface, the tip of the germ tube swells to form an appressorium [4]. Appressorium formation can also be induced on artificial surfaces in the absence of the plant (in vitro) for example on polypropylene foil or in Petri dishes [9, 37]. We found that also glass coverslips are suitable as artificial surface for appressorium formation. This enables high resolution imaging of any actin configuration present in the appressoria. First we focused on the early stages of appressorium formation, and more specifically when the expanding hyphae grew against the coverslip. In these hyphae, we consistently (*n* = 15) observed an aster-like actin cable configuration while contact with the coverslip was established (Fig. 5). As clearly shown in a side view (Fig. 5c), this burst of F-actin is visible at the point where the hypha touches the coverslip. The fluorescence intensity of the aster-like structures is relatively high and at the contact point it is in the same range as the fluorescence of actin filament plaques (Fig. 5d). When followed over time, we observed centrifugally propagating waves of higher intensity of Lifeact-eGFP fluorescence that originated from the center of the aster-like structure (Video S3). We have observed similar propagating waves of fluorescence in the tip of growing hyphae that we interpreted as waves of polymerization [21]. Upon contact with the coverslip, the tips of the hyphae swelled slightly, although the swelling in young appressoria was less prominent than in fully grown appressoria (Fig. 6). In fully grown appressoria the aster-like structure disappeared and the actin organization resembled that of non-growing hyphae, consisting of actin filament cables and actin plaques that were evenly distributed along the cell cortex [21]. In addition, as in non-growing hyphae, a nucleus frequently resided in the vicinity of the hyphal tip (*n* = 10). Rotation of 3D projections of the *Z*-stacks (Fig. 6c) revealed that all of fully grown appressoria were in direct contact with the coverslip, confirming that physical contact is required for appressorium formation. When we monitored the appressoria over time, we could indeed confirm that they had stopped growing (Video S4). Occasionally we observed a new outgrowth emerging from fully grown appressoria that had the actin organization of a regular growing hypha in which actin plaques were absent from the apex (Video S5).

### Transient actin accumulation in *P. infestans* during host cell penetration

In general it is challenging to study the dynamics of an infection process at the microscopic level. In the case of *P. infestans*, zoospores and plant tissue have to be brought together on a microscope slide and have to be kept in optimal conditions for at least several hours. Moreover, actual penetration of the host has to occur close to the coverslip to allow high resolution imaging, and in addition, the host tissue has to be as thin as possible to be able to follow the infection process. We developed an experimental set-up that enabled us to visualize the interaction between *P. infestans* and its host at the microscopic level over time. This set-up makes use of gas-permeable bio-foil slides in which plant cells grown in suspension are co-cultivated with *P. infestans.* In this study, we made use of two plant cell lines, namely the tomato MsK8 suspension cells [29] and the tobacco BY-2 suspension cells [38]. The tomato MsK8–*P. infestans* pathosystem has been developed and optimized in our laboratory, and MsK8 cells turned out to be a very suitable host for *P. infestans*. On the other hand, BY-2 cells display less autofluorescence and are therefore more suitable for imaging than MsK8 cells. However, the infection rates that we obtained with BY-2 cells were extremely low and thus we only used MsK8 cells.

**Fig. 7:**
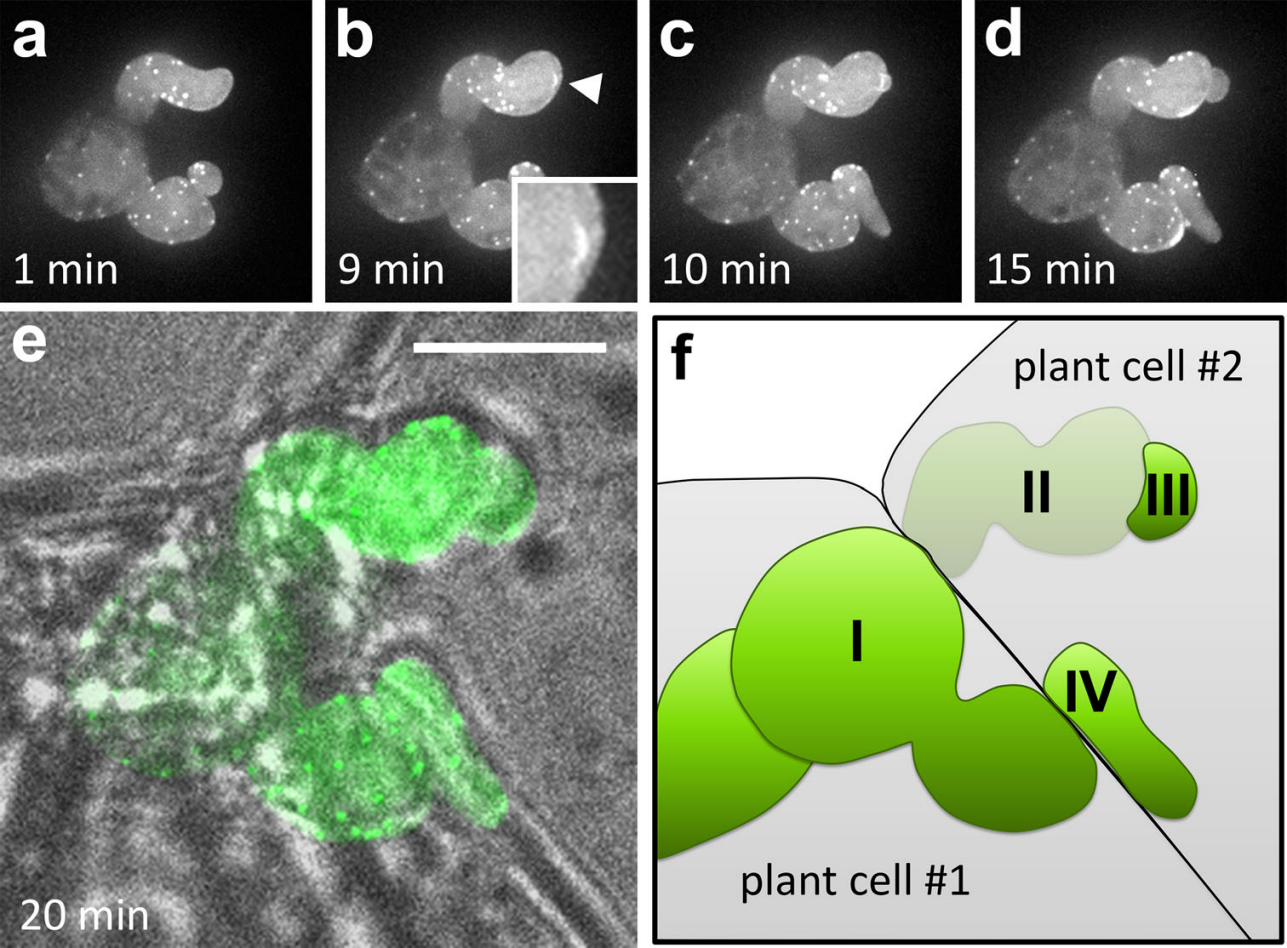
Localization of actin in a *P. infestans* hypha penetrating a MsK8 tomato cell. Maximum intensity *Z*-projections of Lifeact-eGFP fluorescence over time at 1 (**a**), 9 (**b**), 10 (**c**), 15 (**d**) and 20 min (**e**) from the start of the imaging. At *t* = 9 (**b**) penetration takes place. A transient accumulation of F-actin, enlarged in the *inset* and indicated by an *arrowhead*, marks the moment of plant cell penetration. **e** shows the overlay of the bright field and the Lifeact-eGFP fluorescence at 20 min. **f** Schematic overview of **e**. (I) Hypha growing in plant cell #1. (II) Hypha exiting plant cell #1 and growing outside the plant cells before entering. (III) Plant cell #2. (IV) New outgrowth of hypha (I) crossing the cell wall between plant cell #1 and #2. *Bar* 10 μm

We first focused on the moment of penetration of a MsK8 cell by a *P. infestans* hypha (Fig. 7a–f). Zoospores of *P. infestans* were added to MsK8 cells in a bio-foil slide, so that infection could be followed over time. It was challenging to predict when a hypha was about to penetrate a cell and to have this event in focus at the right time. Also, the image quality was reduced when compared to images of hyphae that grow against the coverslip. The large distance between most infection events and the coverslip caused an increased scattering of photons thus resulting in less clear images. Nevertheless, we were able to detect a transient accumulation of F-actin precisely at the penetration point. We examined several penetration events and in all cases we observed this transient accumulation (Fig. 7b; Movie S6; *n* = 4).

### The actin organization in invasive hyphae is indistinguishable from that in hyphae grown in medium

**Fig. 8:**
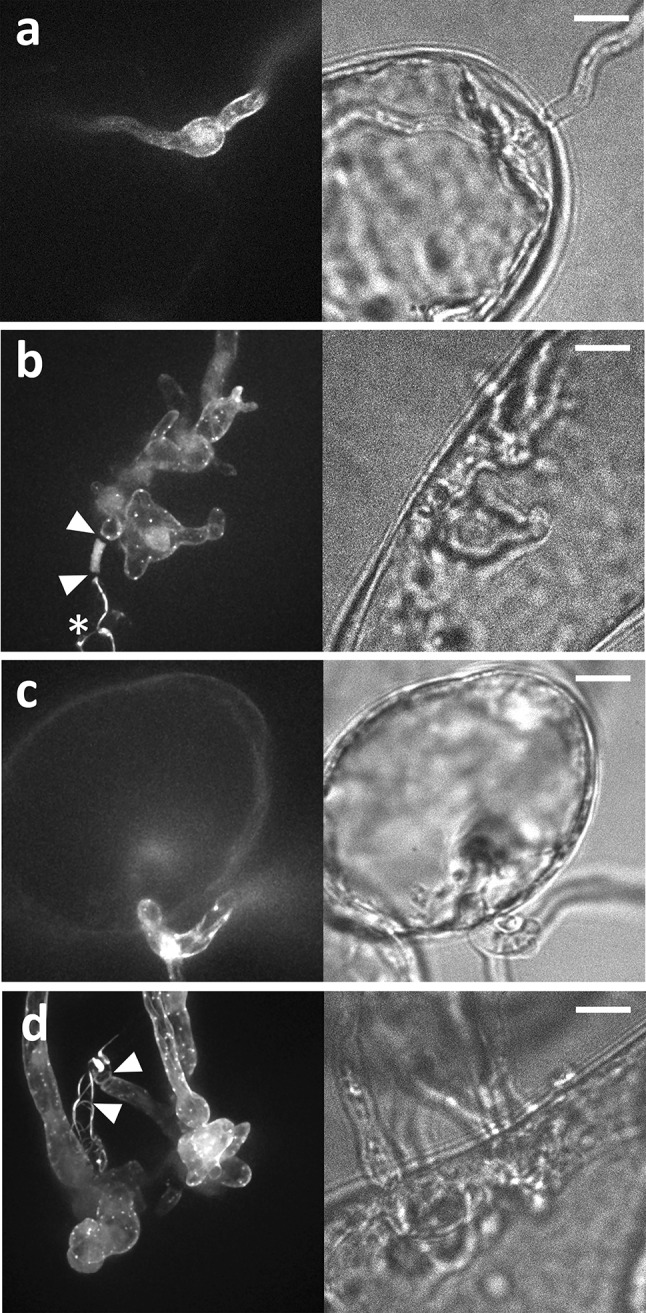
Actin organization in *P. infestans* hyphae that have invaded MsK8 tomato cells. Images show the actin organization in different hyphal structures that develop within the MsK8 cells. **a** Growth directly through a plant cell without branching within the cell. **b** Hypha that has grown directly through a plant cell while producing irregularly shaped branches. **c** A hyphal side branch that has arrested growth briefly after the plant cell penetration. **d** A hyphal side branch that has produced irregularly shaped outgrowths within the plant cell after penetration. In **b** and **d** *arrowheads* point to plugs. In **b** the *asterisk *marks the cyst from which the hypha has emerged. The *right panels* of the images show the bright field and the *left panels* show the fluorescent channel. *Bars* 10 µm

When MsK8 cells were exposed to *P. infestans* zoospores in a bio-foil slide, two types of infections were observed: (1) *P. infestans* hyphae that directly penetrate MsK8 cells, and that exit the cell to continue further growth in the medium (Fig. 8a, b); and (2) *P. infestans* hyphae that penetrate MsK8 cells only after hyphal branching (in the medium) outside cells (Fig. 8c, d). Both infection types were observed frequently. Also in this set-up, cell wall plugs were detected in the germinated cysts similar to those described above when cysts were germinating in the absence of plant cells. However, cell wall plugs were only formed before plant cell penetration and were never observed in hyphae growing inside or emerging from a plant cell (Fig. 8b, d). Hyphae growing outside MsK8 cells had a similar actin organization as hyphae cultured in vitro (previously described in [21]), namely actin plaques in non-expanding parts of the hyphae and actin cables with a net-longitudinally orientation to the length axis of hyphae. A similar actin organization was observed in hyphae that were skewering MsK8 cells (Fig. 8a, b). Also penetration structures of the other type of infection showed similar actin organization as in normal growing hyphae, i.e., with actin filament cables and plaques evenly distributed over the cortex of the cell (Fig. 8c–d). Thus, the actin organization of hyphae that penetrate MsK8 cells did not differ from that of hyphae growing in between cells or in the absence of host cells.

## Discussion

Previously, we have presented live cell imaging of the actin cytoskeleton in in vitro grown hyphae of *P. infestans* and revealed that actin plaques represent novel oomycete-specific actin configurations [21]. Here we have focused on the actin cytoskeleton in *P. infestans* in processes related to plant infection, including germ tube growth from encysted zoospores, appressorium formation, and plant cell penetration, and identified additional novel actin configurations. We detected that actin filament accumulation correlates with cell wall plug formation in germ tubes, and aster-like actin configurations that mark the barrier that appressoria encounter when attempting to penetrate. This aster-like actin configuration is prominently visible during the establishment of contact between an appressorium and the coverslip surface, and a similar transient accumulation of F-actin is also observed during plant cell penetration.

Why do *P. infestans* germlings form cell wall plugs? Generally, oomycetes are known as aseptate organisms. However, for a few oomycetes it has been shown that they have the ability to form septa that compartmentalize hyphae, i.e., *Peronospora tabacina, Pseudoperonospora cubensis* and *Pseudoperonospora humuli* [7]. In addition, in some oomycete species cell wall structures have been reported that are deposited in response to wounding and during sporangium formation and function as sealant [6, 7]. However, the plugs deposited in germinated cysts, also called false septa [9], are different from the wall material deposited after hyphal structural damage. In hyphae from the smut fungus *Ustilago maydis* [39], so-called retraction septa are deposited that, similar to the plugs that we observed in *P. infestans*, seal off the basal part of the hypha after the depletion of cytoplasm. Also in angiosperm pollen tubes, plugs that seal off compartments are deposited and, similar to the plugs in *P. infestans*, pollen tube plugs expand centrifugally, i.e., from the periphery of the tube or hypha towards the central axis [30]. In pollen tubes the plugs consist of callose [40–42]. Once these callose plugs are formed the apical part of the pollen tube can advance independently of the pollen grain and the initial tube, and this reduces the risk of damage, limits the cell volume, and allows growth over longer distances [42, 43]. The retraction septa in *U. maydis* seem to fulfill a similar function [39]. Mutants that are unable to form retraction septa produce much less appressoria and show severely reduced virulence and lower rates of plant penetration [44]. We hypothesize that, similar to callose plugs in pollen tubes and retraction septa in smut fungi, plugs in *P. infestans* are deposited as a response to limitations in resources. Although this is supported by the finding that plugs in *P. infestans* hyphae are only deposited in hyphae emerging from cysts and not in hyphae emerging from sporangia, more research is needed to determine to what extent plug formation in *P. infestans* affects virulence.

The accumulation of F-actin associated with the leading edge of the extending plug that we observed suggests that the actin cytoskeleton is involved in plug deposition. In theory this could be tested by disrupting the actin cytoskeleton and analyze the effect on plug deposition. However, disruption of the actin cytoskeleton by actin depolymerizing drugs also causes inhibition of growth and cytoplasmic streaming [20, 21], making it impossible to distinguish between cause and consequence: is the effect on plug formation caused by the actual actin depolymerisation or indirectly by inhibition of growth and cytoplasmic streaming resulting from actin depolymerisation? Occasionally, some actin plaques remained associated with the cell cortex in the distal part of the hypha after plug deposition. These plaques showed a transient increase in fluorescence whereupon they disappeared. Since the intensity of surrounding actin cables did not change it is likely to be a plaque-specific process. It could for example be due to stabilization of the actin associated with the plaques that may initially cause Lifeact-eGFP to accumulate whereafter it is outcompeted by binding of endogenous actin binding proteins, or to a localized, transient pH increase (>pH 10), that causes eGFP to become more brightly fluorescent [45, 46]. Alternatively, a rapid pulse of actin polymerization followed by complete depolymerizaton of plaques may explain the transient increase in fluorescence. From previous studies, we know that actin plaques are unique oomycete-specific actin structures. In growing hyphae they have a relatively long lifetime and tend to be highly immobile [21]. Here, we showed that during plug formation their lifetime is shorter and that they are more dynamic. As yet, we have no clue what the composition is of actin plaques and how dynamic this composition is. Are the plaques in hyphae the same as the plaques associated with plug formation and what exactly determines their behavior? Isolating the actin plaques, with the aim to identify proteins associated with F-actin, would be the logical next step toward identifying their function and may help to increase our insight in plaque formation and in their role of the overall actin cytoskeleton in *P. infestans*.

When germlings face a physical barrier (in our experimental set-up a coverslip) we observed the assembly of actin that resulted in an aster-like structure at the point of contact. We hypothesize that the observed aster-like actin configuration in appressoria of *P. infestans* is induced by physical contact. Also during penetration of tomato MsK8 suspension cells we consistently observed a transient accumulation of F-actin that was reminiscent of the aster-like structures observed in appressoria at the contact point with the coverslip (Fig. 5). However, the prominence of the actin structures in the in vivo infection system, so during penetration of MsK8 cells (Fig. 7), was much lower in comparison to the actin asters in appressoria formed on glass. This reduced prominence might reflect a correlation between surface strength and the amount of actin that is recruited to breach the surface. In the rice blast fungus *Magnaporthe oryzae*, a torodial actin filament network has a function in the assembly of a septin diffusion barrier. This barrier is essential for increasing the pressure in the appressorium to the level that is required for plant cell penetration [26]. Also in the maize pathogen *Colletotrichum graminicola* Lifeact-GFP accumulates in appressoria at the spot where the penetration peg will form [47]. Compared to fungal appressoria, *P. infestans* appressoria are understudied. It is not known to what extent pressure is important for penetration and how much pressure a *P. infestans* appressorium can handle. Moreover, *P. infestans* lacks genes encoding septins, the GTP-binding proteins that are conserved in many eukaryotes, and form protein complexes that are considered to be part of the cytoskeleton. So besides the shape also the envisioned composition of such a diffusion barrier, if it exists, will differ from the one present in *M. oryzae.* The location and organization of the aster-like actin configuration in *P. infestans* appressoria suggests that it may have a function in cargo transport rather than in assembling or supporting a diffusion barrier. This hypothetical function in cargo transport is supported by the fact that the center of the actin aster is the exact spot from where plant cell penetration is initiated.

In this study, we have identified two novel actin configurations in *P. infestans* and both associate with structures that are important for plant infection. First, we found an accumulation of actin during cell wall plug formation. These plugs allow a hypha emerging from a cyst to extend its range while searching for a suitable entry point on the plant surface. Second, we found an actin network in the appressorium, the infection structure from which a penetration peg is formed that pierces the plant cells. More knowledge about these structures and the role of actin and the novel oomycete-specific actin configurations in assembling these structures may be a first step toward identifying agents that disrupt the assembly processes. The strategy to first pinpoint potential oomycete-specific targets and then develop methods to interfere with these targets holds promise for oomycete disease control in the future.

## Electronic supplementary material

Below is the link to the electronic supplementary material.

Supplementary material 1 Video S1 A *P. infestans* hypha during plug formation. Lifeact-eGFP accumulates at the site of plug formation. The Lifeact-eGFP signal stays associated with plug formation site until the process is completed. Hyphal tip is located outside the field of view. Structure on the left of the plug is an adjacent appressorium. *Z*-stacks were collected every 30 s and are displayed as maximal intensity projections. Total video duration is 31 min. *Bar* 5 µm. Avi file: 5 frames/s (AVI 192 kb)

Supplementary material 2 Video S2 An appressorium formed by *P. infestans* that grows against the coverslip shows a Lifeact-eGFP signal at the contact point. *Z*-stacks were collected every 30 s and are displayed as maximal intensity projections. Total video duration is 15 min. *Scale bar* 5 µm. Avi file; 5 frames/s (AVI 206 kb)

Supplementary material 3 Video S4 A non-expanding *P. infestans* appressorium expressing Lifeact-eGFP located against the cover slip. Video also shows the start of the formation of a plug just below the appressorium. *Z*-stacks were collected every 30 s and are displayed as maximal intensity projections. Total video duration is 15 min. *Scale bar* 5 µm. Avi file; 5 frames/s (AVI 83 kb)

Supplementary material 4 Video S4 A *P. infestans* appressorium expressing Lifeact-eGFP showing a new outgrowth. *Z*-stacks were collected every 60 s and are displayed as maximal intensity projections. Total video duration is 12 min. *Scale bar* 5 µm. Avi file; 3 frames/s (AVI 205 kb)

Supplementary material 5 Video S6 *P. infestans* expressing Lifeact-eGFP penetrating a plant cell. *Z*-stacks were collected every 60 s and are displayed as maximal intensity projections. Total video duration is 20 min. *Scale bar* 10 µm. Avi file; 3 frames/s (AVI 82 kb)

Supplementary material 6 Video S6 A *P. infestans* hypha in a stage just prior to plug formation which starts 7 min after this video ends. The cytoplasm is retracting and Lifeact-eGFP labeled plaques disappear with the retracting cytoplasm. Hyphal tip and position of plug formation are located outside the field of view. *Z*-stacks were collected every 30 s and are displayed as maximal intensity projections. Total video duration is 15 min. *Bar* 5 µm. Avi file: 5 frames/s (AVI 130 kb)
